# 

**Published:** 1809-02

**Authors:** 


### NEW MEDICAL PUBLICATIONS.

Observations on an Eruptive Disease which has lately occurred in the town of Sherborne, Dorset, after Vaccination. In a letter to a Friend. By Richard Pew, M. D. of Sherborne, Member of the Royal Medical and other Societies, Edinburgh. 1s. 6d.

The New Practical Family Physician, or Improved Domestic Medical Guide. Containing a very plain Account of the Causes, Symptoms, and Method of curing every Disease incident to the Human Body, with the most safe and rational Means of preventing them, by an improved Plan of Regimen, Air, and Exercise, adapted for the use of private Families. By Thomas Furlong Churchill, M. D. 8vo. 11s. bound.

Reports on the Effects of a peculiar Regimen on Schirrous Tumours and Cancerous Ulcers. By William Lambe, M. D. 8vo. 5s.

The Physician's Vade Mecum. By Robert Hooper, M. D. small 8vo. 5s.

### To CORRESPONDENTS.

Dr. Bellamy's Paper has been received. His MS. on "Military Hospitals," was left at the Bar of Mascall's Hotel, by Dr. Bradley's own hand, with a particular charge respecting the care of it.

Communications are received from Dr. Kinglake and Mr. Hall.

